# Detection of calcitonin gene-related peptide based on increased antigen-driven interaction with antibody variable regions

**DOI:** 10.3389/fbioe.2024.1395330

**Published:** 2024-05-31

**Authors:** Yueqing Cheng, Yujie Gao, Shengshuo Zhang, Yujie Zou, Guangwei Zhao, Liyuan Zheng, Binghui Hou, Mei Li, Jinhua Dong

**Affiliations:** ^1^ School of Life Science and Technology, Shandong Second Medical University, Weifang, China; ^2^ School of Rehabilitation Sciences and Engineering, University of Health and Rehabilitation Sciences, Qingdao, China; ^3^ School of Basic Medical Sciences, Shandong University, Jinan, China; ^4^ Department of Neurology, the Affiliated Hospital of Qingdao University, Qingdao, China; ^5^ International Research Frontiers Initiative, Tokyo Institute of Technology, Yokohama, Japan

**Keywords:** migraine, CGRP, detection, antibody, open sandwich ELISA

## Abstract

**Introduction:** Calcitonin gene-related peptide (CGRP) is involved in trigeminal neuralgia and migraine, and measuring the CGRP concentration in the serum is crucial for the early prediction of these conditions. Current methods for CGRP detection are primarily radioimmunoassay, which needs radioactive substances and enzyme-linked immunosorbent assays (ELISAs) which need long detection time and some have a narrow detection range.

**Methods:** The genes of anti-CGRP antibody variable regions were cloned into pDong1 vector to obtain pDong1/Fab-CGRP, with which phage-Fab was prepared, and the concentration of CGRP was detected by competitive ELISA. The pDong1/Fab-CGRP was modified to obtain pDong1/OS-CGRP, with which the co-expression solution containing phage-displayed heavy chain variable fragments (phage-V_H_) and light chain was obtained. CGRP was detected by OS-ELISA based on phage-V_H_, antibody light chain, and anti-light chain antibody. The V_L_ gene was cloned into the pMAL vector to obtain pMAL-V_L_ (CGRP), with which maltose binding protein fused with V_L_ (MBP-V_L_) was prepared. CGRP was detected by OS-ELISA employing MBP-V_L_ and phage-V_H_.

**Results:** OS-ELISAs that measure the CGRP concentration by quantifying the interaction between variable regions were investigated. OS-ELISA using phage-V_H_ and secreted light chains in the same culture system exhibited a limit of detection (LOD) of 0.05 nM, offering higher sensitivity than competitive assay with an LOD of 0.75 nM, whereas using phage-V_H_ and separately prepared MBP-V_L_ exhibited an LOD of 0.15 nM and a broader detection range of 0.15–500 nM than competitive ELISA, whose detection range was 0.75–10 nM.

**Discussion:** The combination of the two OS assays achieved high sensitivity and a broad detection range for CGRP, which may have significance in clinical applications.

## 1 Introduction

Migraine is a common recurring disorder characterized by severe headaches on one or both sides of the head and is often accompanied by symptoms such as nausea, vomiting, and sensitivity to light and sound (Welch, 1997). Migraine is classified by the World Health Organization as the sixth most disabling disease worldwide, and it imposes a significant burden on social and economic productivity. Migraine is categorized into episodic and chronic forms, with episodic migraine often progressing to chronic migraine as the symptoms worsen. Individuals with chronic migraines have a significantly higher likelihood of developing cardiovascular diseases, respiratory disorders (Wang et al., 2020), anxiety, depression (Duan et al., 2023), and chronic pain (Buse et al., 2010). Approximately 15% of adults worldwide suffer from migraines, with a prevalence rate of 9.3% in China and a male-to-female ratio of 1:3 (Yu et al., 2012; Yu et al., 2014). In addition, migraines are associated with stroke to some extent. Perioperative stroke is a common complication, and individuals with migraines are at a significantly higher risk of experiencing ischemic stroke during the perioperative period compared to those without migraines (Bousser and Welch, 2005; Kurth et al., 2012). The probability of transient ischemic attacks is twice as high in women with migraines than in those without migraines. Carotid artery dissection is a primary cause of ischemic stroke in young people and is more common in patients with migraines. Migraine also increases the likelihood of hemorrhagic stroke (Chang et al., 1999; Tzourio et al., 2002). Given the lack of preventive treatments, migraines are ranked sixth among disabling diseases globally, imposing a significant burden on society, the economy, and individuals.

**FIGURE 1:**
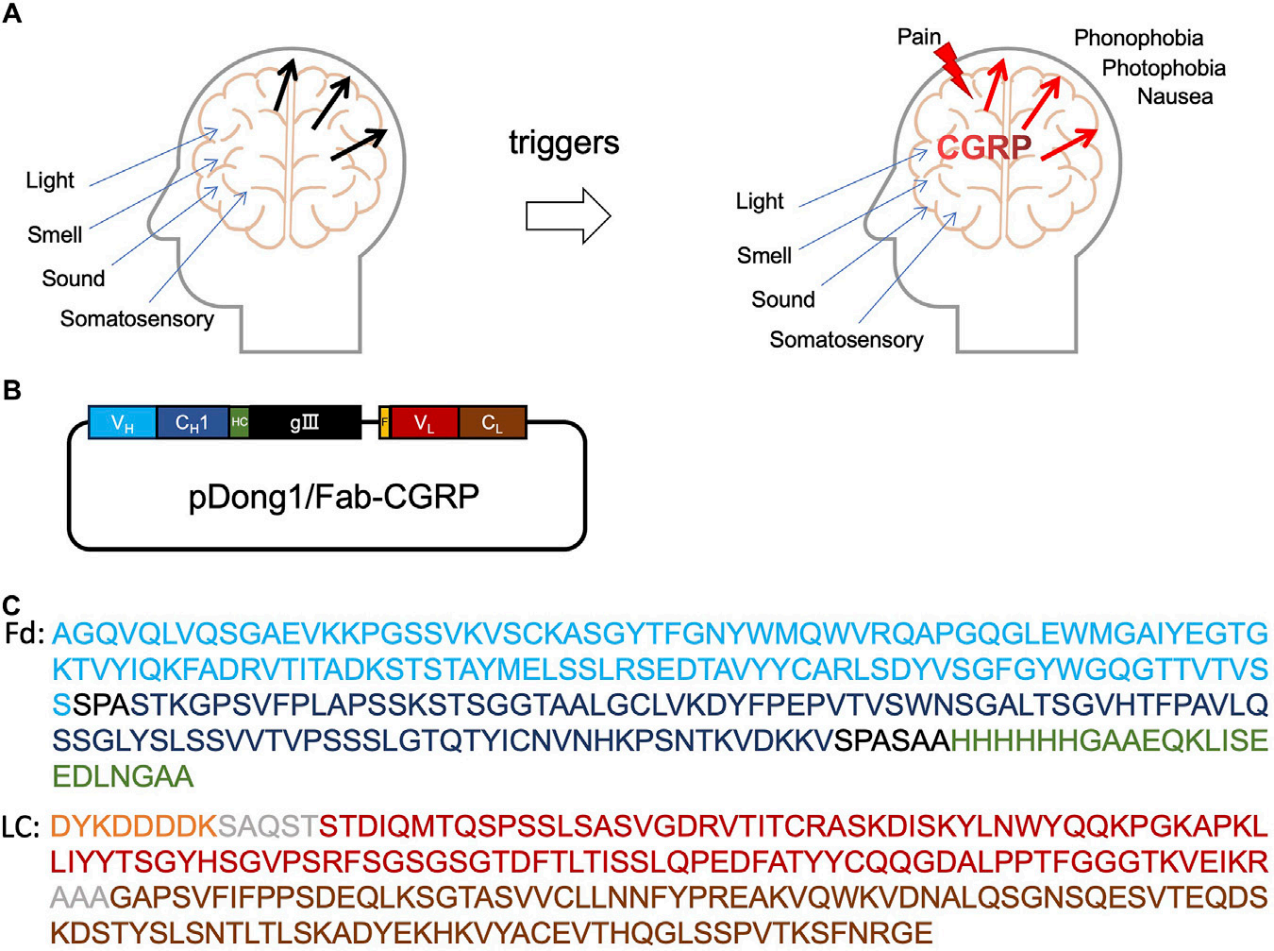
Calcitonin gene-related peptide (CGRP) and anti-CGRP antibody. Role of CGRP in headache **(A)**, the construction of pDong1/Fab-CGRP for the phage display of anti-CGRP antibody **(B)**, and the amino acid sequences of the designed Fd and light chain of the phage-displayed antibody **(C)**. V_H_: heavy chain variable region of antibody (blue color); C_H_1: constant region one of antibody heavy chain (dark blue); HC: his-tag and c-myc tag (green color); gIII: gIII protein of phage; F, flag-tag (orange); V_L_, light chain variable region (red color); C_L_, light chain constant region (brown color); Fd, V_H_-C_H_1 regions of heavy chain.

The pathophysiology of migraine is not fully understood, but the trigeminal vascular theory holds a dominant position in migraine pathogenesis. According to the trigeminal vascular theory, migraines are triggered by the trigeminal vascular system activation, resulting in the release of various vasoactive substances, with calcitonin gene-related peptide (CGRP) playing a major role (Figure 1A) (Lassen et al., 2002; Schou et al., 2017). Studies in the early 1990s confirmed that blood CGRP levels increase during migraine attacks. Nitric oxide-induced migraine attacks also result in elevated CGRP levels in both the blood and saliva. Injecting CGRP into patients can lead to migraine attacks (Alpuente et al., 2022). CGRP is a multifunctional neuropeptide comprising 37 amino acids (Supplementary Figure S1) and is widely distributed in various systems of the human body, being primarily found in the dorsal root ganglia and trigeminal nerves (Durham, 2006). CGRP does not directly mediate pain but sensitizes other pathways to pain. Inflammatory and neuropathic pain states are associated with increased CGRP levels, which lead to increased pain perception and are involved in trigeminal neuralgia and migraine attacks (Benemei et al., 2009). During migraine attacks, CGRP levels in the peripheral blood of patients with migraines are significantly higher than those in healthy individuals (Cernuda-Morollon et al., 2013). Therefore, accurate and rapid measurement of CGRP levels in the body holds potential for predicting trigeminal neuralgia and migraines.

Current methods for detecting CGRP are based on the use of anti-CGRP monoclonal antibodies. In 1991, a radioimmunoassay for CGRP detection was developed, which involves complex procedures including serum extraction, gel chromatography, high-performance liquid chromatography, a 4-day incubation with specific antibodies, and a 2-day incubation with tracers (Schifter, 1991). In 1999, a sandwich method was devised for CGRP detection using two antibodies. Two hybridoma cell lines were generated through the fusion of mouse spleen cells and myeloma cells to produce the monoclonal antibodies mAb-CGRP-83 and mAb-CGRP-72, which were used in the assay. The minimum detectable concentration using this method was close to 2 pg/mL; however, the total testing time was extended to 18 h (Frobert et al., 1999).

The Open Sandwich ELISA (OS-ELISA) technique is an immunological assay based on the interaction of variable antibody regions (Ueda et al., 1996). The principle of the OS-ELISA is that the variable regions of the heavy chain (V_H_) and light chain (V_L_) of the antibody are separated in the absence of an antigen. When an antigen is present, the antigen, V_H_, and V_L_ bind together, resulting in different absorbance values depending on the antigen concentration, allowing for specific antigen detection. OS-ELISA only needs one antibody, being simpler than Sandwich ELISA, and small molecules or peptides can be detected non-competitively. This technology has been widely applied for detecting various substances such as thyroid hormones (Islam et al., 2011), clenbuterol hydrochloride (Cong et al., 2019), aflatoxins (Su et al., 2023), and microcystin-LR (Chen et al., 2020). In the present study, an OS-ELISA method was employed to develop a bioassay for CGRP, offering the advantages of high sensitivity and a wide detection range, which are of great importance for CGRP measurement and migraine diagnosis.

## 2 Materials and methods

### 2.1 Materials

The genes for V_H_ and V_L_ of the anti-CGRP antibody were designed based on previously reported sequences (Benschop et al., 2014), and synthesis was carried out by Shanghai Sangon Biotechnology (Shanghai, China). Plasmid extraction reagents, including the DiaSpin column-based plasmid DNA miniprep kit, DipSpin column-based DNA gel recovery kit, and EZ-10 column-based polymerase chain reaction (PCR) product purification kit, were purchased from Shanghai Sangon Biotechnology (Shanghai, China). *Escherichia coli* DH5α was used for gene cloning and plasmid amplification, since it lacks immunity to exogenous DNA and therefore does not cleave exogenous DNA, allowing it to stably maintain exogenous DNA in its cells. *E. coli* TG-1, obtained from GE Healthcare (Tokyo, Japan), was used for phage display, which can suppress amber mutations, thus serving as a host for plasmids and bacteriophages carrying amber codons. *E. coli* BL21 (DE3) was used for protein expression, since it lacks the genes for the proteases lon and ompT, which facilitates the purification of exogenous proteins. The DH5α and BL21 (DE3) were purchased from Kangti Life Technology (Guangzhou, China). Restriction enzymes were purchased from New England BioLabs (NEB, Beijing, China). The primers used in this study were obtained from Shanghai Sangon Biotechnology. Biotin-CGRP was synthesized by LifeTein, LLC (Hillsborough, NJ, United States). Unless otherwise specified, all other reagents were purchased from Sigma-Aldrich or GenScript Biotechnology (Shanghai, China).

### 2.2 CGRP antibody gene amplification

The anti-CGRP antibody gene was synthesized and inserted into a pUC57 vector. Using the pUC57 vector as a template, the V_H_ and V_L_ genes were amplified by PCR with polymerase KOD-Plus-Neo (TOYOBO, Japan) using the primer pairs NcoICGRPVHback/XhoICGRPVHfor and SalCGRPVLback/NotCGRPVLforII. The amplification program used three steps repeated in 30 cycles: denaturation at 94°C for 30 s, annealing at 55°C for 30 s, and extension at 72°C for 60 s. The PCR products were recovered after being separated by agarose gel electrophoresis. The primers used in this study are listed in Table 1.

**TABLE 1:** Primers used in this study.

Primer name	Sequence (5′-3′)	Length (bp)
NcoICGRPVHback	CCG​GCC​ATG​GCC​GGT​CAG​GTG​CAA​C	25
XhoICGRPVHfor	TGA​GCT​CGA​GAC​AGT​TAC​GGT​AGT	24
SalCGRPVLback	CTC​AGT​CGA​CTT​CGA​CGG​ACA​TTC​AG	26
NotCGRPVLforII	GCC​TGC​GGC​CGC​GCG​TTT​GAT​CTC​GAC​TTT	30
M13Rv	CAGGAAACAGCTATGAC	17
pHENseq	CTATGCGGCCCCATTCA	16
pMALCGRPVLSalFor	CGC​GTC​GAC​TTC​GAC​GGA​CAT​TCA​GAT​GA	29
pMALCGRPVLNotRev	GAT​GTG​CGG​CCG​CGC​GTT​TGA​TCT​CGA	27

### 2.3 Construction of pDong1/Fab-CGRP

The pDong1 vector (Dong et al., 2009) and recovered V_H_ genes were digested with *Nco*I and *Xho*I at 37°C for 5 h. After digestion, agarose gel electrophoresis was performed, and the target fragments were recovered using a gel recovery kit. The recovered V_H_ fragment was then ligated with the pDong1 vector using Ligation high Ver.2 (TOYOBO Bio) at 16°C for 2 h. The ligation product was transformed into *E. coli* DH5α and spread on Luria-Bertani agar (LB) plates containing 100 μg/mL of ampicillin (LBA). The plates were incubated at 37°C for 12 h, and single colonies were selected after 12 h. These colonies were subjected to colony PCR using the primers M13rv, pHENseq, and Easy-Load PCR Master Mix (Beyotime Biotechnology, Shanghai, China). Colonies with successful PCR results were selected and cultured in 4 mL of an LB liquid medium at 37°C for 12 h. The plasmid DNA was extracted and used for downstream experiments.

Plasmid pDong1, including the V_H_ gene (pDong1-V_H_) and the pre-amplified V_L_ gene, was subjected to enzymatic digestion using *Sal*I and *Not*I and recovered after agarose gel electrophoresis. The V_L_ gene was then ligated into the recovered pDong1-V_H_ vector using the same method. The ligation product was transformed into *E. coli* DH5α and spread on LBA plates. After incubation at 37°C for 12 h, single colonies were picked, and colony PCR was performed using the primers SalCGRPVLback and NotCGRPVLforII. The colonies with the right DNA insert were cultured in 4 mL of an LB liquid medium at 37°C for 12 h, and plasmid DNA extraction was carried out. The plasmids were sequenced, and those with accurate sequences were designated as pDong1/Fab-CGRP and used for phage display.

### 2.4 Preparation of phage-displayed Fab

The successfully constructed recombinant plasmid pDong1/Fab-CGRP was transformed into *E. coli* TG-1 competent cells for phage display. A TG-1 colony with pDong1/Fab-CGRP was picked and transferred to 2 YT medium with 100 μg/mL of ampicillin and 1% glucose (2YTAG) medium, where they were left to grow overnight. The next day, 40 μL of the overnight culture was transferred to 4 mL of a fresh 2YTAG medium. When the bacterial culture reached an optical density at 600 nm of approximately 0.4, 10^9^ PFU of the KM13 helper phage (Kristensen and Winter 1998) were added. The mixture was incubated at 37°C for 30 min, followed by incubation at 25°C for 10 min. After centrifugation at 3,300 *g* for 10 min at 4°C, the supernatant was removed. A total of 4 mL of the 2YTAG medium containing 50 μg/mL kanamycin (2YTAGK) was added to the remaining pellet. The culture was then incubated at 30°C for 20 h with shaking at 220 rpm. Following incubation, the bacterial culture was centrifuged at 4°C for 15 min at 3,300 g, and the supernatant was collected. To 4 mL of the supernatant, 1 mL of polyethylene glycol 6,000/2.5 M NaCl solution (PEG/NaCl) was added, and the mixture was thoroughly mixed before being placed on ice for 1 h. After the incubation, the mixture was centrifuged at 5,500 *g* for 1 h at 4°C. The supernatant was removed, and the pellet was centrifuged again to eliminate the remaining supernatant. The pellet was suspended in 200 μL of phosphate buffer saline (PBS), and after centrifugation at 11,600 *g* for 10 min at 4°C, the supernatant was collected and stored at −20°C.

### 2.5 ELISA for identifying the antigen-binding activity of phage-displayed Fab

Streptavidin (SA) was coated on microplates at 5 μg/mL and left at 4°C overnight. The next day, 2 μg/mL of biotin-labeled CGRP was added and incubated for 30 min. After blocking with PBS containing 2% skim milk (MPBS) for 2 h, the plates were washed twice with PBS containing 0.5% TWEEN 20 (PBST). Each well was incubated with 10^9^ CFU of phage-Fab at 25°C for 1 h. The microplates were washed six times with PBST, followed by the addition of a 5000-fold diluted anti-M13/HRP antibody in MPBS and incubation at 25°C for 1 h. After six washes with PBST, the 3, 3, 5, 5-tetramethylbenzidine (TMBZ) substrate was added for color development at 37°C. The reaction was terminated with 20% sulfuric acid, and the absorbance at 450 and 630 nm was measured using an iMark™ microplate reader (Bio-Rad).

### 2.6 Competitive ELISA detection of CGRP using phage-displayed Fab

Competitive ELISA was conducted to detect CGRP. SA was coated on microplates at 5 μg/mL, and biotin-labeled CGRP was added the next day at 20 μg/mL, with an incubation time of 30 min. After blocking with MPBS for 2 h, the plates were washed twice with PBST. In each well, CGRP solutions at 0, 0.05 nM, 0.5 nM, 1 nM, 2.5 nM, 5 nM, 10 nM, 25 nM, 50 nM, and 100 nM were added along with phage-Fab, and each well was brought to a total volume of 100 μL with MPBS. The plates were then incubated for 1 h. After six washes with PBST, the assay was performed according to the protocol described in Section 2.5.

### 2.7 Construction of pDong1/OS-CGRP

The C_H_1 gene of pDong1/Fab-CGRP had *Sgr*AI restriction enzyme sites at both the 5′and 3′ends. The pDong1/Fab-CGRP vector was digested with *Sgr*AI at 37°C for 5 h. After digestion, the DNA fragments were recovered and self-ligated with Ligation high Ver.2 at 16°C for 2 h. The ligation product was then transformed into *E. coli* DH5α and plated, followed by incubation at 37°C for 12 h. After 12 h, colony PCR was performed using primers M13rv and pHENseq. Colonies with bands of the correct size were picked after agarose gel electrophoresis and cultured in 4 mL of the LBA medium overnight. The next day, plasmids were extracted and sequenced, and the plasmid with the deleted C_H_1 gene was collected and named pDong1/OS-CGRP.

### 2.8 Preparation of Phage-V_H_ and L chain Co-expression supernatant

pDong1-OS/CGRP was transformed into *E. coli* TG-1 cells, which were then plated. The following day, well-growing single colonies were picked and cultured in 4 mL of the 2YTAG medium at 37°C overnight. The following day, 40 μL of the culture was added to 4 mL of fresh medium and cultured at 37°C. When the OD_600_ reached approximately 0.4, a KM13 helper phage was added, and infection was introduced. The culture was then centrifuged, and the pellet was resuspended in 2YTAGK and cultivated at 30°C for 20 h with shaking at 200 rpm. The culture was taken out and centrifuged at 5,500 g for 30 min at 4°C to obtain the supernatant, which contained the phage-V_H_ and L chains. The co-expression supernatant was subjected to phage titer testing.

### 2.9 Detection of CGRP with phage-V_H_, L chain, and Anti-L chain antibody

Anti-L chain (κ) antibodies (MBL, Beijing) were diluted to a concentration of 2 μg/mL and coated on microplates at 4°C overnight. The following day, the plates were blocked with 3% MPBS for 2 h. After blocking, the plates were washed thrice with PBST. In each well, final concentrations of 0, 0.005, 0.05, 0.5, 5, 50, and 500 nM CGRP were added along with the phage-V_H_ and light chain co-expression supernatant. Instead of CGRP, the C-terminal peptide of human bone Gla protein (BGP-C7; NH_2_-RRFYGPV-COOH) was added to the assay as a negative control. The volume in each well was brought to 100 μL with MPBS, and the plates were left at 25°C for 1 h. After incubation, the plates were washed six times with PBST. Then, a 1:5,000 diluted anti-M13/HRP antibody was added and incubated at 25°C for 1 h. The plate was washed six times with PBST, and the assay was developed using the methods described in Section 2.5.

### 2.10 Construction of pMAL-V_L_ (CGRP)

To detect CGRP without relying on anti-L-chain antibodies, the vector pMAL-V_L_ (CGRP) was constructed to express V_L_ fused with maltose binding protein (MBP-V_L_). The V_L_ gene was amplified by PCR using primers pMALCGRPVLSalFor and pMALCGRPVLNotRev under conditions same to those used for the V_L_ gene amplification. The PCR product was recovered, digested with *Sal*I and *Not*I, ligated to the pMAL-DON vector (unpublished data), which was treated with the same enzymes. The ligation mixture was used to transform *E. coli* DH5α and plated on LBAG plates. After culture, colony PCR was performed using the primers pMALCGRPVLSalFor and pMALCGRPVLNotRev to screen for successful clones. The plasmid with the correct sequence was selected and designated as pMAL-V_L_ (CGRP).

### 2.11 Expression and purification of MBP-V_L_

The pMAL-V_L_ (CGRP) vector was transformed into *E. coli* BL21 (DE3). A single colony was picked and inoculated into 4 mL of an LBA medium, followed by incubation at 37°C for 12 h. A total of 3 μL of this culture was then inoculated into 300 mL of LB liquid medium and cultured at 37°C with shaking at 150 rpm until the OD_600_ reached 0.6. Isopropyl β-D-1-thiogalactopyranoside was then added at a concentration of 0.4 mM, and the culture temperature was reduced to 16°C with shaking at 110 rpm for 20 h. The culture medium was collected and centrifuged at 9,500 *g* for 10 min to remove the supernatant. The bacterial pellet was resuspended in 10 mL of TALON washing buffer. After centrifugation to remove the supernatant, the cells were thoroughly washed with washing buffer. Subsequently, 10 mL of TALON washing buffer was added to completely resuspend the cells, followed by cell disruption. The bacterial lysate was centrifuged at 9,500 g for 20 min, and the supernatant was collected. A total of 300 μL of Ni-NTA Sefinose™ Resin was washed with TALON washing buffer, added to the supernatant of the disrupted cells, and rotated at 4°C for 2 h. The supernatant and Ni-NTA resin were transferred to a purification column. The column was washed with TALON washing buffer to remove impurities, and the target protein was eluted using an elution buffer and analyzed by sodium dodecyl sulfate-polyacrylamide gel electrophoresis (SDS-PAGE).

### 2.12 Preparation of Phage-V_H_ and CGRP detection with MBP-V_L_ and Phage-V_H_

A total of 4 mL of a solution containing phage-V_H_ and L chain was mixed with 1 mL of PEG/NaCl and incubated on ice for 1 h, followed by centrifugation at 5,500 *g* for 1 h at 4°C, and the supernatant was removed. The pellet was suspended in 200 μL PBS, and then centrifugation at 11,600 g for 10 min at 4°C was carried out to recover the supernatant containing phage-V_H_.

MBP-V_L_ was coated on microplates overnight at 20 μg/mL and blocked with 3% MPBS for 2 h after being washed thrice with PBST. After blocking, an MPBS solution containing 10^9^ CFU of phage-V_H_ and CGRP at a series of concentrations (0.005, 0.05, 0.5, 5, 50, and 500 nM) was added to the wells and incubated for 1 h. The microplates were washed six times with PBST, and an anti-M13/-HRP antibody diluted 1:5,000 in MPBS was added and incubated for 1 h. After washing six times, TMBZ was added, and the assay was performed as described in Section 2.5.

### 2.13 Molecular docking

Molecular docking utilized the multimer model in Alphafold v2.3.2 to predict the multimeric structure of the antibody. The parameters used were all default settings. The database versions used were as follows: uniport and uniref90 as of 2023-03-01, pdb_mmcif and pdb_seqres as of 2023-03-03, and the remaining database versions were the default.

### 2.14 Data analysis

In the experiment of obtaining dose-response curves, three samples were set for each concentration, and three parallel experiments were conducted simultaneously to ensure the accuracy of the experiment. Dose-response curves were fitted to a four-parameter equation as shown following using Prism 9 (GraphPad Software, San Diego, CA, United States) and the goodness of fit was evaluated with R squared value.

$$y=a+\frac{b-a}{{\left(1+\frac{x}{c}\right)}^{d}}$$

In the competitive assays, the detection limit (LOD) was defined as the antigen concentration corresponding to the absorbance measured in the absence of antigen, minus three times the error value. In the OS-ELISA method, the LOD was defined as the antigen concentration corresponding to the absorbance measured in the blank plus three times the standard deviation. The half-maximal inhibitory concentration (IC_50_) in the competitive assay was defined as the concentration at which half of the maximum binding was inhibited. The half-maximal effective concentration (EC_50_) of the OS-ELISA method was defined as the antigen concentration at which the maximum value was halved.

## 3 Results

### 3.1 Design and construction of phagemid for displaying Fab

The phagemid pDong1/Fab-CGRP was constructed to display the antigen-binding fragment (Fab) of the anti-CGRP antibody. As shown in Figures 1B,C, the V_H_-C_H_1 gene of the antibody was linked to the gIII gene of the M13 phage with a His-tag and C-myc-tag. This resulted in the V_H_ being displayed on the phage, and His-tag and C-myc-tag could be used for protein detection. The light chain of Fab contained V_L_ and IgG kappa chains with a FLAG tag on the N terminus. Kappa chain and C_H_1 dimerize and result in Fab formation.

**FIGURE 2:**
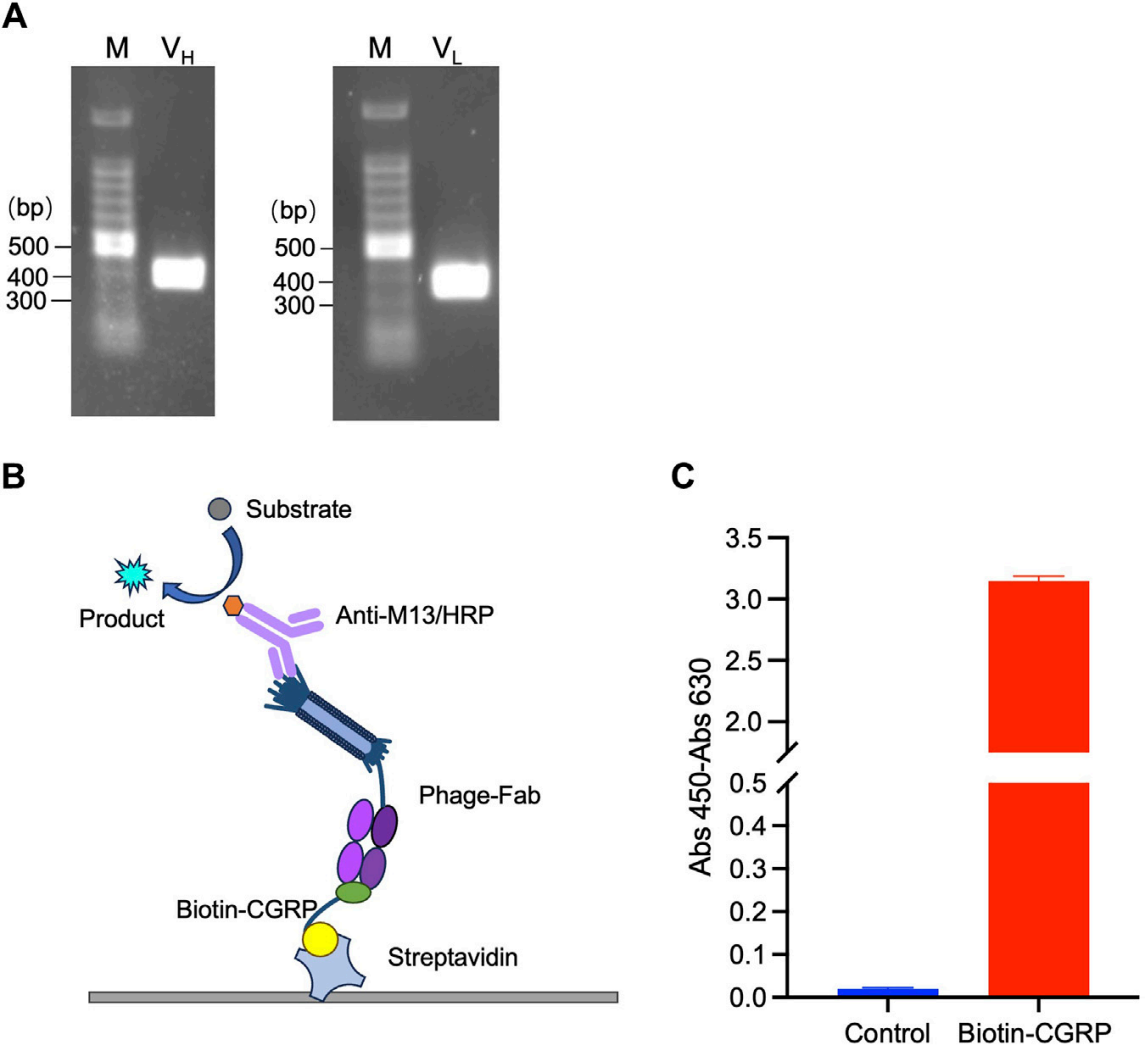
Phage display of antigen-binding fragment (Fab) of the anti-CGRP antibody. Polymerase chain reaction (PCR)-amplified V_H_ and V_L_ genes **(A)**; scheme of sandwich ELISA **(B)** and the results of ELISA for confirmation of antigen-binding activity of phage-displayed Fab **(C)**. The streptavidin-coated plates were used to immobilize biotinylated CGRP (Biotin-CGRP), and detection was achieved by the addition phage-Fab and Anti-M13/HRP.

The V_H_ and V_L_ genes were amplified using PCR and confirmed by agarose gel electrophoresis. As shown in Figure 2A, two distinct bands at the 300-400 bp range were observed, indicating successful amplification of V_H_ and V_L_. The PCR products were recovered and cloned into the pDong1 vector to obtain pDong1-V_H_. V_L_ was cloned into pDong1-V_H_ using a similar approach, resulting in the phagemid vector pDong1/Fab-CGRP, which allows the generation of Fab-displaying phages via *E. coli* TG-1, a suppressor strain.

### 3.2 Detection of Anti-CGRP activity of phage-Fab

To validate the binding activity of Fab to the CGRP antigen, ELISA was performed following the principle illustrated in Figure 2B. Because CGRP is a small molecule that is difficult to immobilize directly onto a plate, an immobilization step with streptavidin was used. The streptavidin-coated plates were further used to immobilize biotinylated CGRP (Biotin-CGRP), and detection was achieved by the addition of MPBS containing 10^9^ CFU/mL of phage-Fab. The results are shown in Figure 2C. The absorbance of samples containing CGRP was 3.15, whereas that of samples without CGRP was 0.02, indicating that the Fab displayed on the phage surfaces had a high affinity for the CGRP antigen.

### 3.3 Competitive ELISA for CGRP detection

**FIGURE 3:**
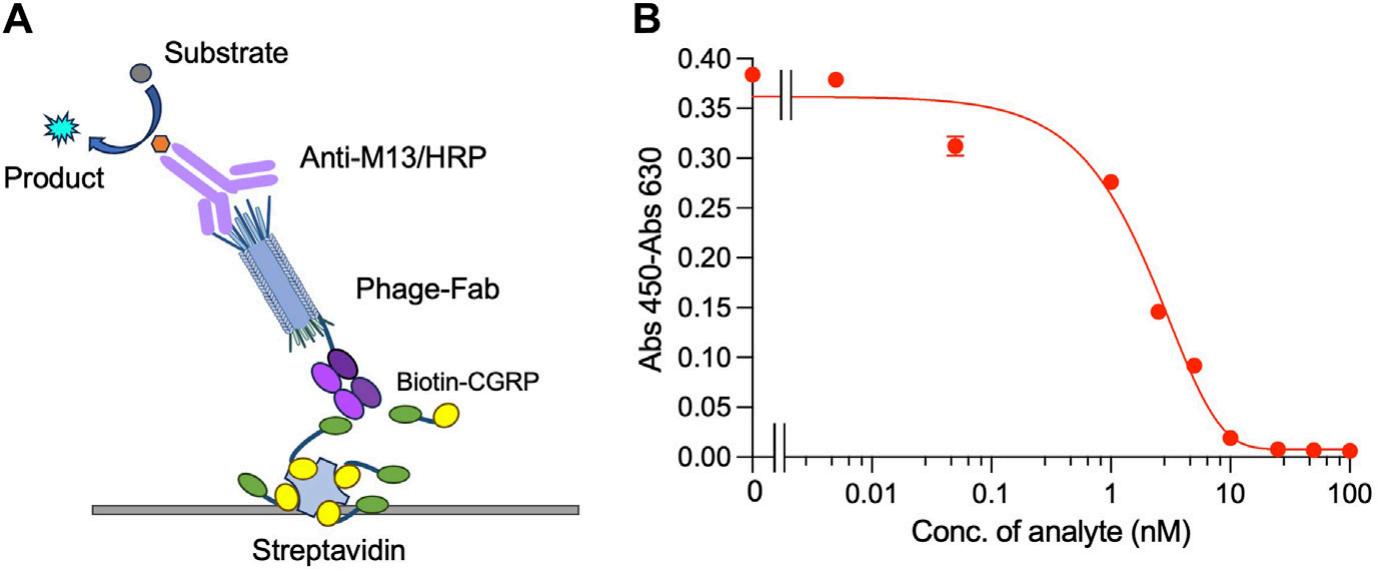
Competitive ELISA for detecting CGRP. Scheme **(A)** and the dose-response curve **(B)** of the assay. Biotin-CGRP, captured by streptavidin coated on a microplate, binds to phage-Fab in a competitive manner with free CGRP. Then, detection is carried out through anti-M13/HRP, achieving quantitative detection of free CGRP.

Indirect competitive ELISA was performed on plates containing immobilized streptavidin and biotin-labeled CGRP to detect CGRP, as shown in Figure 3A. When the free CGRP concentration in the solution was low, Fab was displayed on the phage bound to the immobilized Biotin-CGRP, resulting in a higher absorbance. As the free Biotin-CGRP concentration increased, phage-Fab was competitively bound to free Biotin-CGRP, leading to reduced binding to the plate-immobilized Biotin-CGRP and decreased absorbance. Using different concentrations of CGRP for competitive ELISA experiments, based on the measured data, we obtained a dose-response curve with an *R* ^2^ value of 0.984. The LOD for CGRP was calculated to be 0.75 nM, with a detection range of 0.75–10 nM, and the IC_50_ was 2.07 nM (Figure 3B).

### 3.4 Construction of pDong1/OS-CGRP

**FIGURE 4:**
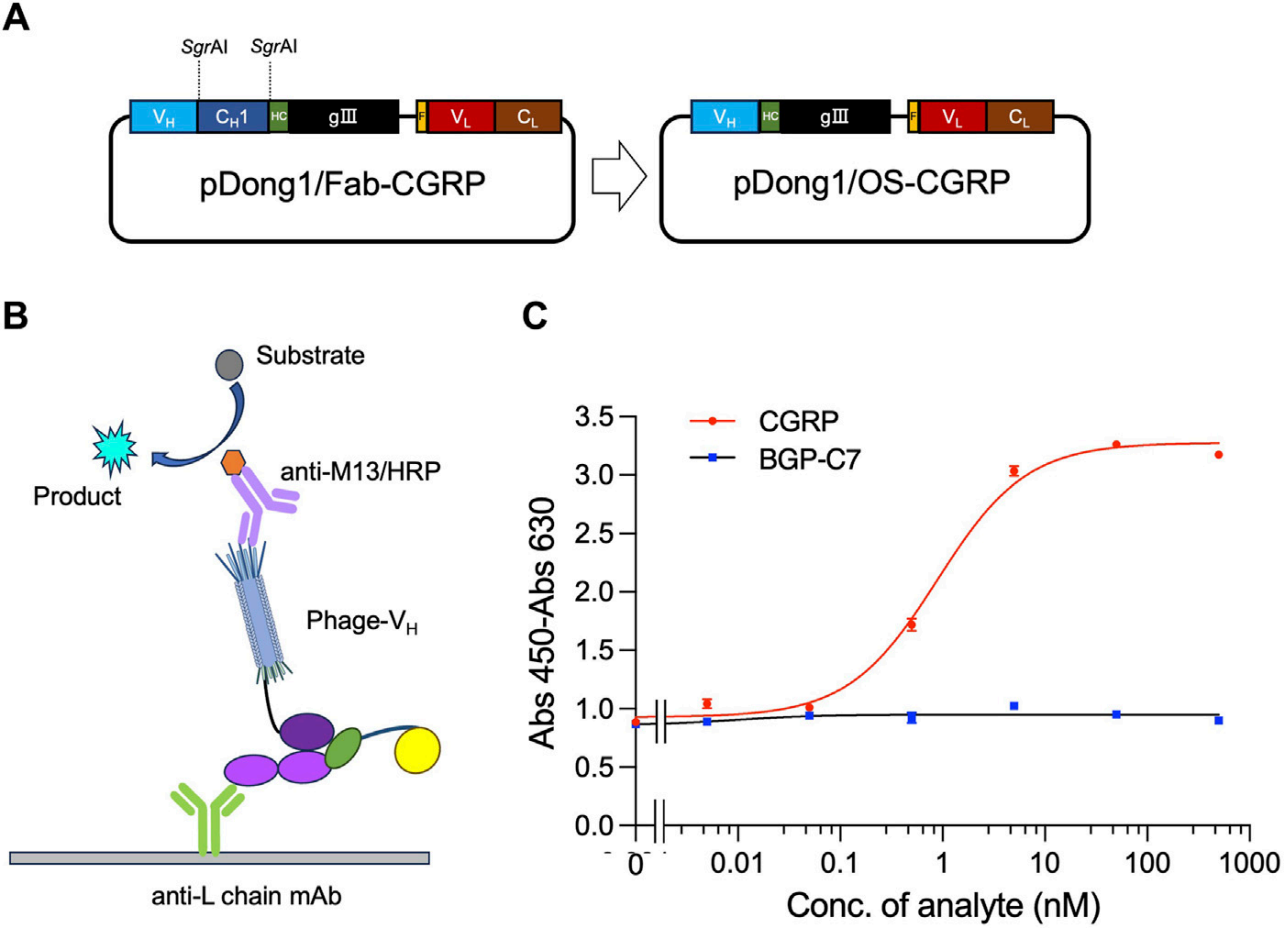
Detection of CGRP with phage-displayed V_H_ and expressed light chain prepared in the same system. Construction of the vector for preparing phage solution **(A)**; scheme for OS ELISA with phage and light chain prepared in the same system **(B)**; dose-response curve of the assay **(C)**. BGP-C7: C terminal peptide of bone gla protein, which was used as a negative control. The antibody light chain is captured by anti-L chain antibody coated on a microplate. It binds with CGRP together with phage-V_H_, forming a trimer. The quantity of phage-V_H_ in the trimer is detected using anti-M13/HRP, achieving quantitative detection of CGRP.

The OS-ELISA employs separate V_H_ and V_L_. To prepare elements for performing the OS-ELISA, the phagemid pDong1/OS-CGRP was prepared. We used the pDong1 system to delete the C_H_1 open reading frame (ORF), resulting in the display of only the V_H_ on the phage. In the Fab display system, Fab is formed through the interaction between C_H_1 and C_L_ of the light chain. Because there is no C_H_1 gene in pDong1/OS-CGRP, we co-secreted the light chain and phage-V_H_ into the culture supernatant separately, allowing for OS-ELISA. Self-ligation was performed after processing with *Sgr*AI (Figure 4A). The colony PCR results indicated that the sizes of the PCR products for clones 1, 2, and three were 650 bp, which was smaller than those of the PCR products obtained without removing the C_H_1 plasmid, confirming a successful construction (Supplementary Figure S2). This streamlined approach enabled rapid assessment of the interaction between V_H_ and V_L_ and its antigen dependency without the need for recloning segments. One single colony was picked and cultured in 4 mL of liquid LBA medium for 12 h, followed by plasmid extraction and sequencing. Sequence results without errors were designated as pDong1/OS-CGRP.

### 3.5 OS-ELISA for CGRP detection

An OS-ELISA with phage-V_H_, light chain antibody, and anti-L chain antibody was performed, as illustrated in Figure 4B. A mixture of co-expression solutions with different CGRP concentration gradients was used. In the presence of the CGRP antigen, the light chain and phage-V_H_ exhibited apparent antigen dependency, resulting in stronger signals as the antigen concentration increased. Therefore, the final absorbance increased with increasing antigen concentration (Figure 4C). As the CGRP concentration in the samples increased, the signal gradually increased and stabilized. In contrast, the detection signal of the negative control sample using BGP-C7 did not increase, indicating that the specificity of the detection technique and the antibody displayed a clear antigen-dependent signal increase. The LOD and EC_50_ for CGRP detection were 0.05 nM and 0.89 nM, respectively, with a detection range of 0.05–10 nM, determined using a dose-response curve with an *R* ^2^ value of 0.993. OS-ELISA exhibited higher sensitivity and a broader detection range than competitive ELISA. From these results, we conclude that removing the C_H_1 region of antibodies did not affect their specificity.

### 3.6 Expression and purification of MBP-V_L_

To simplify the OS detection technique, another OS-ELISA assay platform with phage-V_H_ and MBP-V_L_ was evaluated.

**FIGURE 5:**
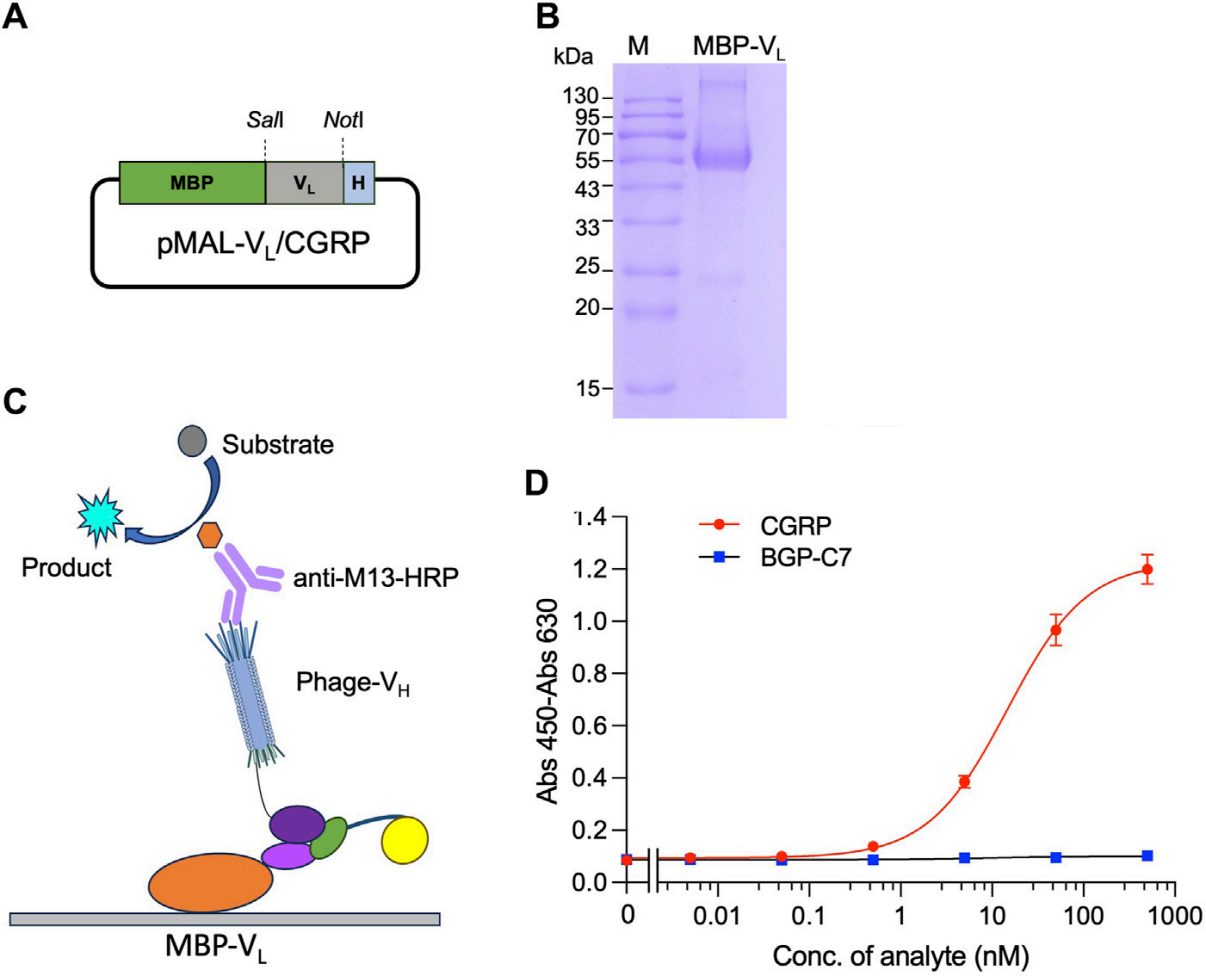
Detection of CGRP with phage-displayed V_H_ and separately expressed V_L_ fused with maltose binding protein (MBP-V_L_). Construction of the vector for expression of MBP-V_L_ **(A)**; SDS-PAGE analysis of purified MBP-V_L_ **(B)**; scheme for OS immunoassay with phage-V_H_ and MBP-V_L_ **(C)**; and dose-response curve for CGRP detection **(D)**. MBP-V_L_ coated onto the microplate binds with CGRP together with phage-V_H_, forming a trimer. The quantity of phage-V_H_ in the trimer is detected using anti-M13/HRP, achieving quantitative detection of the antigen.

First, plamid pMAL-V_L_(CGRP) for the expression of MBP-V_L_ was constructed. The V_L_ gene was amplified by PCR from pDong1/Fab-CGRP. The PCR product was subjected to agarose gel electrophoresis, and a distinct band at the 300–400 bp range (Supplementary Figure S3A) was observed, indicating successful amplification of the V_L_ gene. The gene was cloned into the pMAL vector, and the presence of V_L_ was confirmed by colony PCR (Supplementary Figure S3B) and sequence analysis. The successfully constructed pMAL-V_L_ (CGRP) (Figure 5A) was used to transform *E. coli* BL21 (DE3) cells, and protein expression was induced with IPTG. MBP-V_L_ was purified using a Ni^2+^ column and analyzed using SDS-PAGE. As shown in Figure 5B clear band was observed with a molecular weight of 55 kDa, corresponding to MBP-V_L_ with a theoretical molecular weight of 55 kDa. The SDS-PAGE results also demonstrated the purity of MBP-V_L_ of 93%, which was further utilized in OS-ELISA.

### 3.7 OS-ELISA based on phage-V_H_ and MBP-V_L_

After coating with MBP-V_L_, a mixture of phage-V_H_ and different CGRP concentrations was added. In the presence of the CGRP antigen, MBP-V_L_ and phage-V_H_ would bind together. The higher the antigen concentration, the higher the absorbance values (Figure 5C). As the CGRP concentration in the sample increased, the signal gradually increased and tended to stabilize, generating a dose-response curve as shown in Figure 5D. The LOD and EC_50_ for this assay were 0.15 nM and 14.6 nM, respectively, with a wide detection range of 0.15 nM–500 nM, determined using a dose-response curve with an *R* ^2^ value of 0.996. The detection signal for the negative control sample BGP-C7 did not increase, indicating the specificity of the assay.

### 3.8 Structural analysis

**FIGURE 6:**
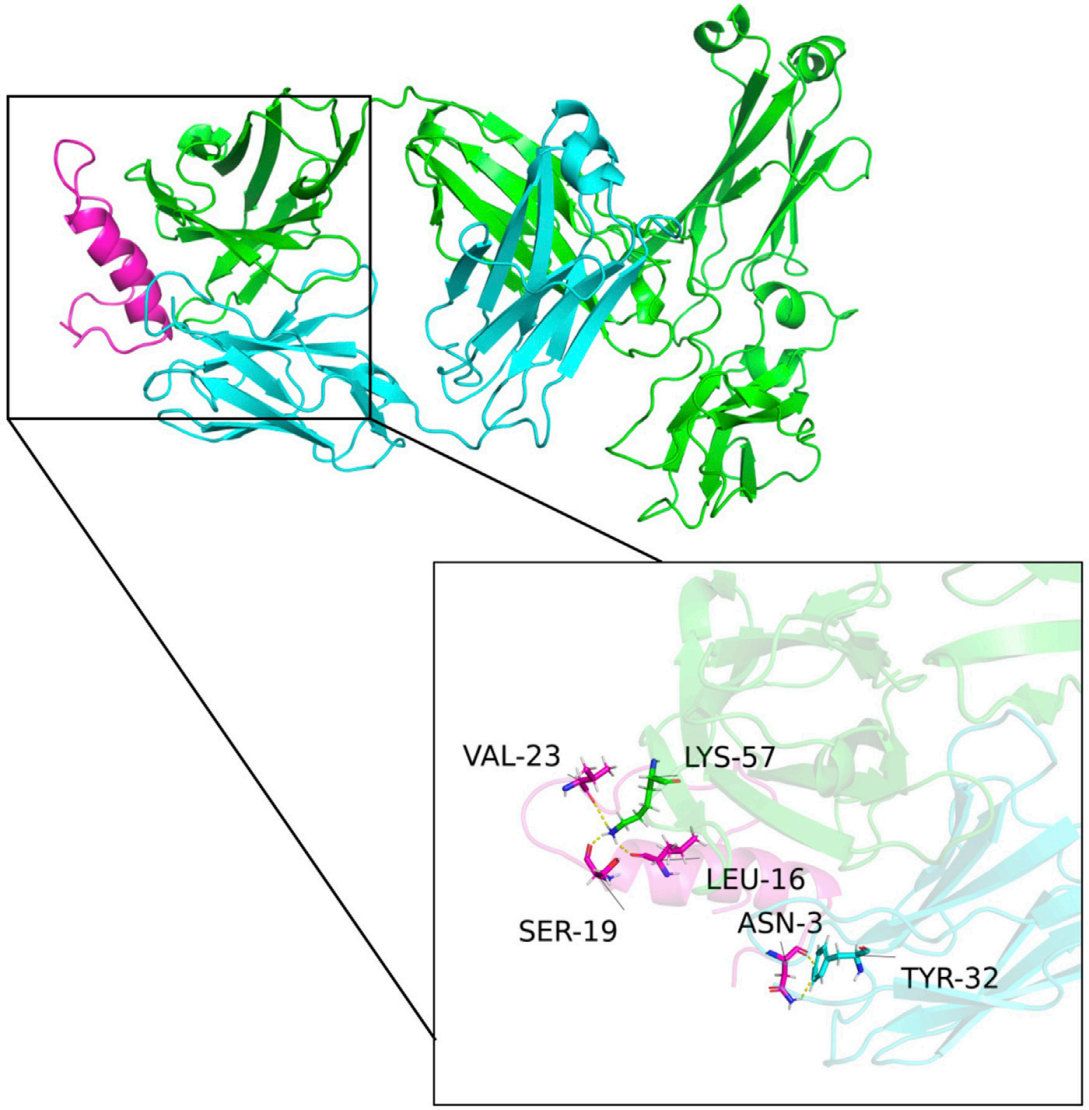
Structural model of the antibody and CGRP binding. Green, blue, and purple represent the antibody heavy chain variable region, the light chain variable region, and the CGRP antigen, respectively. Isoleucine at position 16 (LEU-16), serine at position 19 (SER-19), and valine at position 23 (VAL-23) of CGRP formed hydrogen bonds with lysine at position 57 (LYS-57) of the heavy chain variable region. Asparagine at position 3 (ASN-3) of CGRP formed a hydrogen bond with tyrosine at position 32 (TYR-32) of the light chain variable region.

The predicted multimeric structures were analyzed and identified amino acid pairs between proteins that can interact to form hydrogen bonds as shown in Figure 6. Specifically, isoleucine at position 16 (LEU-16), serine at position 19 (SER-19), and valine at position 23 (VAL-23) of CGRP formed hydrogen bonds with lysine at position 57 (LYS-57) of the heavy chain variable region. Asparagine at position 3 (ASN-3) of CGRP formed a hydrogen bond with tyrosine at position 32 (TYR-32) of the light chain variable region. The formation of these hydrogen bonds enhances the interaction between the antibody V_H_ and V_L_.

## 4 Discussion

This study describes a CGRP detection method based on the interaction between the V_H_ and V_L_ regions of an anti-CGRP antibody. The region of an antibody that interacts with the antigen epitope is the complementary determining region (CDR), which is located in the variable region of the antibody. OS immunoassays are based on the interaction of V_H_ and V_L_. In the absence of CGRP, the V_H_ and V_L_ of an anti-CGRP antibody are prone to separate, whereas when CGRP is present, it binds to the CDRs of the variable regions. The interaction between V_H_ and V_L_ is enhanced with CGRP binding to form trimers. By measuring the number of trimers formed, the antigen can be quantitatively detected. OS-ELISA is a non-competitive detection method for small molecules, and thus, it is not limited by the affinity of the antibody, in contrast with the traditional competitive detection that has a relatively narrow detective range. In addition, the heavy chain and the light chain variable regions in OS-ELISA are from the same antibody, and they only occupy one epitope on the surface of the antigen; therefore, there is no molecular size limit for the test sample. Even small molecules with a molecular weight <1,000 can be detected by OS-ELISA. It offers advantages such as simplicity, short detection time, quick procedure, and broad detection range. It does not require large instruments such as those necessary for HPLC analysis, thus avoiding delays in the detection of disease markers and the weakening of antigen activity caused by prolonged experimental durations.

In the present study, two types of OS immunoassays were investigated. Those using a phage display of antibody heavy chain variable fragments (phage-V_H_) and secreted light chains in the same culture system exhibited the highest sensitivity, whereas those using phage-V_H_ and separately prepared MBP fused light chain variable regions exhibited a broader detection range. Notably, different antibodies with varying amino acid sequences exhibited diverse conformations. The interaction between the V_H_ and V_L_ differed for each antibody; not all antibodies are suitable for the OS-ELISA system. In this study, the phage display system pDong1 was used, which enabled the rapid evaluation of variable fragments suitable for OS-ELISA.

Compared with traditional methods, the proposed assays achieved a broader detection range and shorter detection time. However, the sensitivity of the assay was 0.05 nM, whereas the CGRP levels in an individual are between 0.008 and 0.071 nM. Therefore, the assay sensitivity needs to be improved to meet clinical needs. As an approach, the original interaction between V_H_ and V_L_ could be decreased by induction of some mutants (Liang et al., 2021). The assay time may be optimized, and to further simplify the detection process, homogeneous assays such as Quenchbody technology (Abe et al., 2011; Dong and Ueda, 2021) that can detect CGRP in real time may be developed. For future medical applications, the stability of V_H_ and V_L_ and the suitability of the buffer may need further investigation.

A fast and accurate method for detecting CGRP is crucial for the timely diagnosis, prevention, and treatment of migraine, ultimately reducing the disability rate. The OS immunoassay described in this study may provide a novel approach for detecting CGRP levels in patients with migraine and determining migraine causes.

## Data Availability

The original contributions presented in the study are included in the article/Supplementary Material, further inquiries can be directed to the corresponding authors.
