# Supplementary material for: Detection of calcitonin gene-related peptide based on increased antigen-driven interaction with antibody variable regions
Source: Front Bioeng Biotechnol. 2024 May 31;12:1395330. doi: 10.3389/fbioe.2024.1395330 (PMC11176539; doi:10.3389/fbioe.2024.1395330)
Supplement: Supplementary file 1 [file DataSheet1.docx]

**Supplementary Materials for**

**Detection of Calcitonin Gene-related Peptide Based on Increased Antigen-Driven Interaction of Antibody Variable Regions**

Yueqing Cheng^a,b^, Yujie Gao^a^, Shengshuo Zhang^a^, Yujie Zou^a^, Guangwei Zhao^b,c^, Liyuan Zheng^b^, Binghui Hou^d^, Mei Li^a*^, and Jinhua Dong ^b, e*^

^a^*School of Life Science and Technology, Shandong Second Medical University, Weifang, China*

^b^*School of Rehabilitation Sciences and Engineering, University of Health and Rehabilitation Sciences, Qingdao, China*

*^c^School of Basic Medical Sciences, Shandong University, Ji’nan, China*

^d^*Department of Neurology, the Affiliated Hospital of Qingdao University, Qingdao, China*

^e^*International Research Frontiers Initiative, Tokyo Institute of Technology, Yokohama, Japan*

*Corresponding authors

Jinhua Dong, jhdong@uor.edu.cn

Mei Li, limei@wfmc.edu.cn

**Phage Titer Determination**

A 1 μL aliquot of the original phage solution was diluted a thousandfold with PBS to obtain a dilution factor of 10^3^. Then, 10 μL of the 10^3^-fold diluted phage solution was added to 100 μL of PBS to achieve a dilution factor of 10^5^. Subsequently, 10 μL of the 10^5^-fold diluted phage solution was added to 90 μL of PBS to create a dilution factor of 10^6^. Finally, 10 μL of the 10^6^-fold diluted phage solution was added to 90 μL of PBS to yield a dilution factor of 10^7^.

From the 10^3^-fold diluted phage solution, 10 μL was taken and diluted in 100 μL of Escherichia coli TG-1 bacterial culture with an OD of 0.3, resulting in a 10^5^-fold diluted TG-1 bacterial culture. Similarly, 10 μL of the 10^5^-fold, 10^6^-fold, and 10^7^-fold diluted phage solutions were each diluted in 100 μL of OD 0.3 TG-1 bacterial culture. This process generated TG-1 bacterial cultures with phage dilution factors of 10^5^, 10^6^, 10^7^, and 10^8^. From each of these four bacterial cultures, 10 μL of the culture was plated and incubated for 12 hours, after which colony numbers was counted.


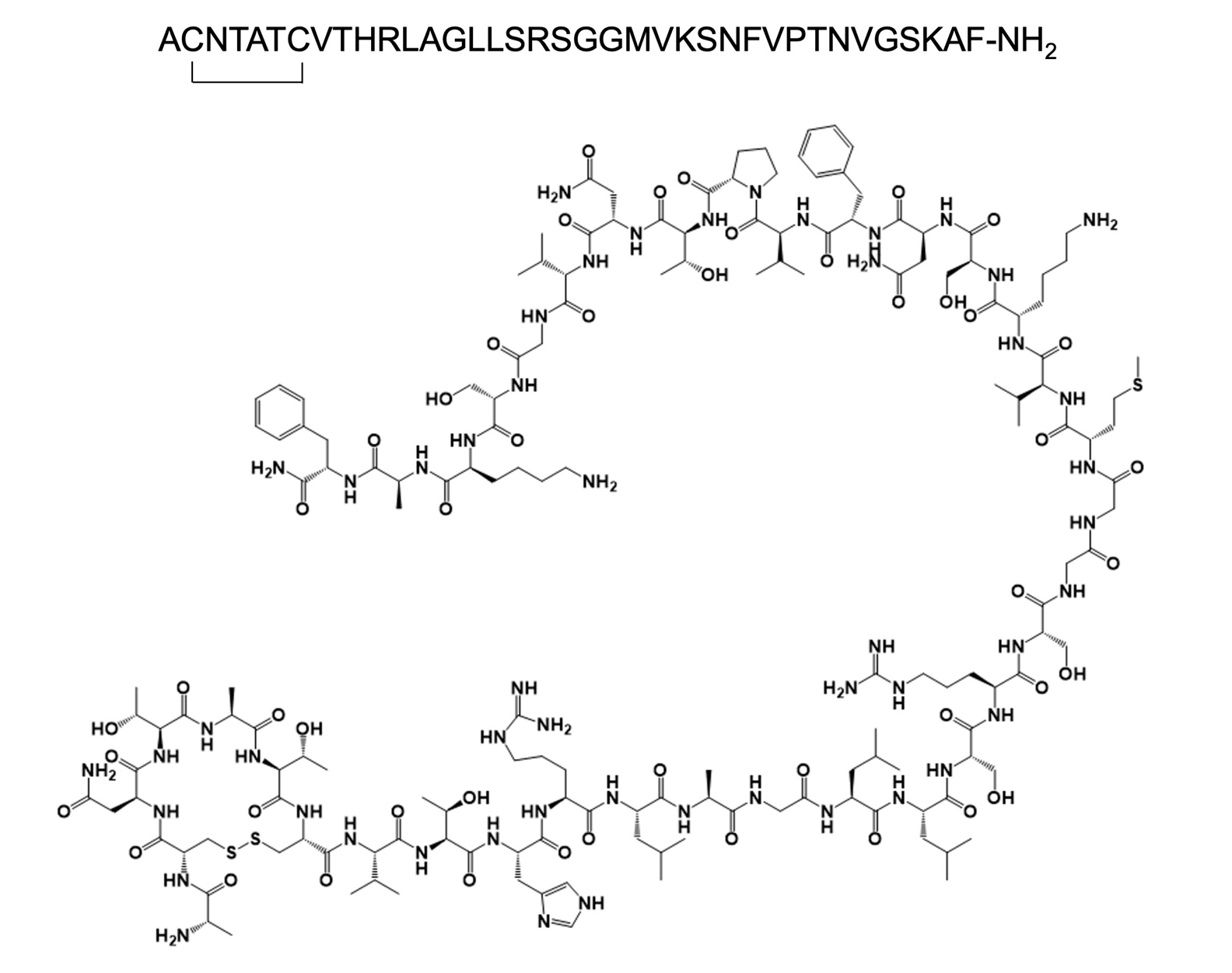


**Fig. S1** Amino acid sequence of CGRP.


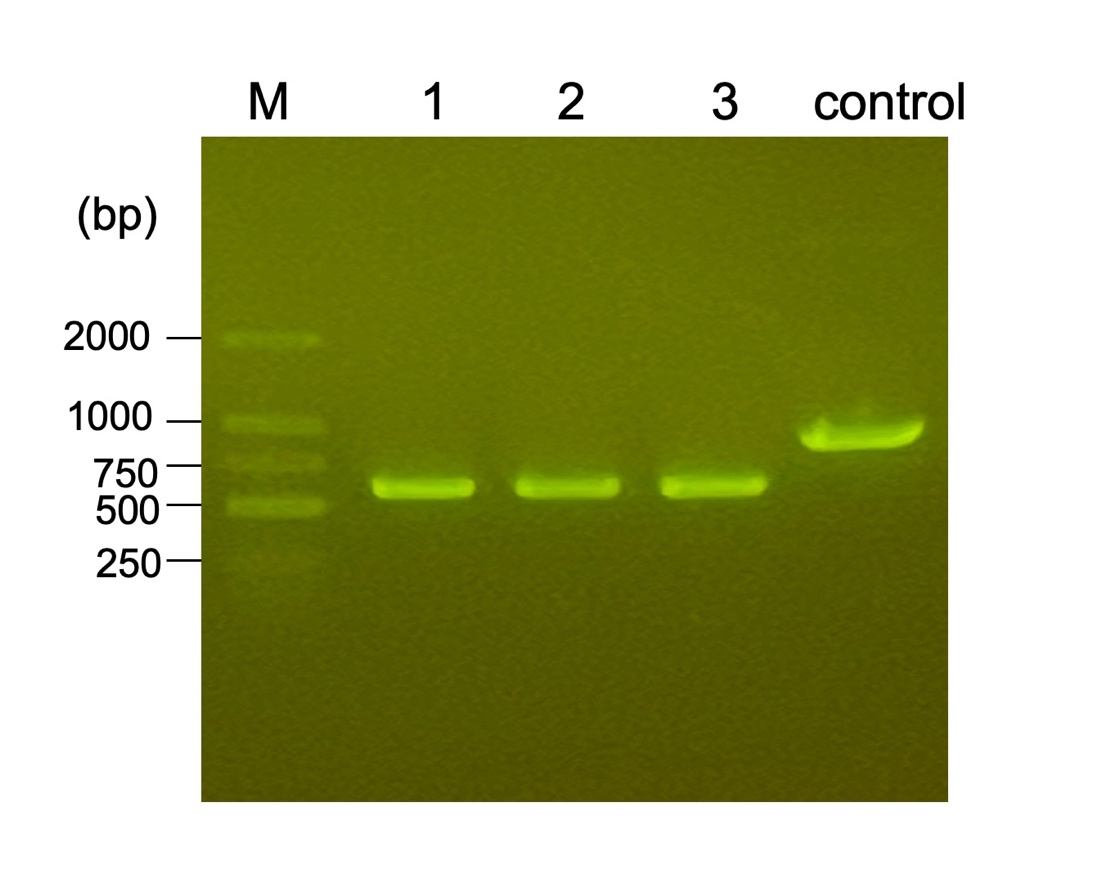


**Fig. S2** Results of colony polymerase chain reaction for screening positive colony containing right plasmid pDong1/OS-CGRP.


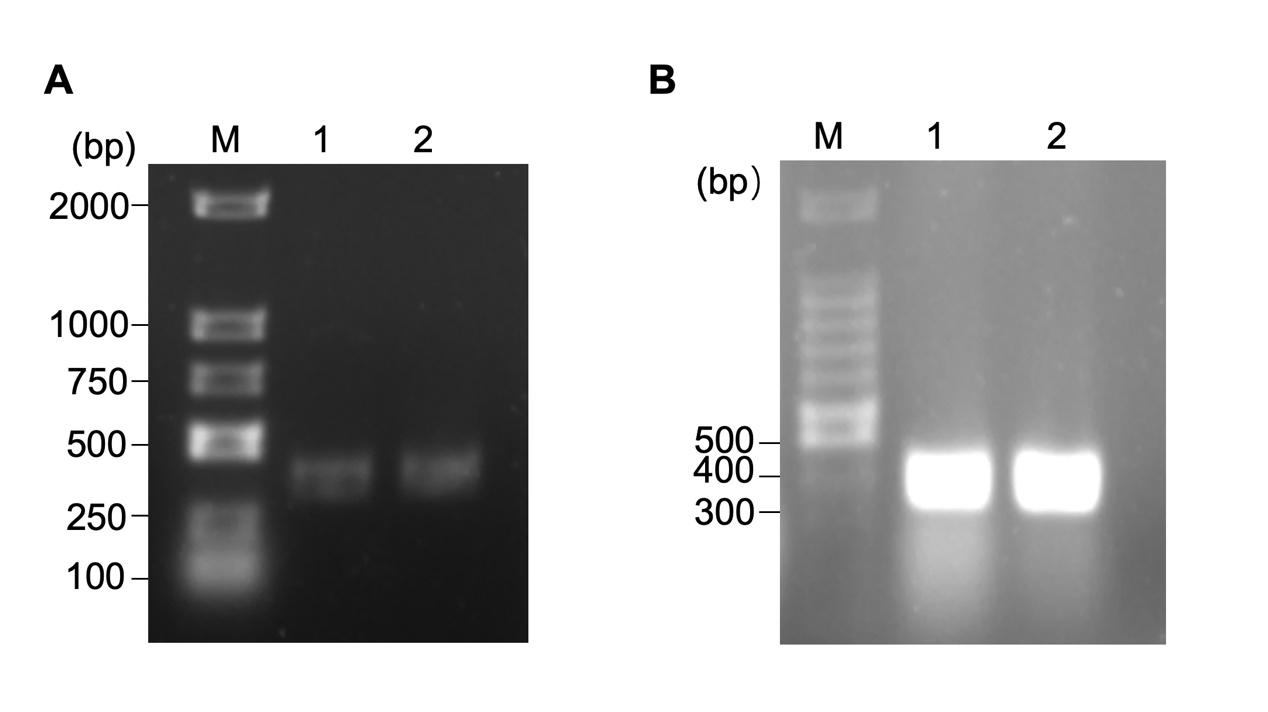


**Fig. S3** Amplified V_L_ (A) and colony PCR for identify positive clone (B) for construction of pMAL-V_L_(CGRP).
